# Switching of the harmonic order in free-electron lasers by controlling the density modulation of an electron bunch

**DOI:** 10.1107/S1600577518008937

**Published:** 2018-08-03

**Authors:** Norihiro Sei, Hiroshi Ogawa, QiKa Jia

**Affiliations:** a Research Institute for Measurement and Analytical Instrumentation, National Institute of Advanced Industrial Science and Technology, 1-1-1 Umezono, Tsukuba, Ibaraki 305-8568, Japan; b National Synchrotron Radiation Laboratory, University of Science and Technology of China, Jinzhai Road 96, Hefei, Anhui Province 230026, People’s Republic of China

**Keywords:** free-electron lasers, higher harmonic oscillation, optical klystron, effective gains, inverse Compton scattering X-rays

## Abstract

It was demonstrated that harmonic order in free-electron laser (FEL) oscillations could be switched by adjusting the dispersive gap of the optical klystron ETLOK-III in the storage ring NIJI-IV. The effective gains for the fundamental and third-harmonic FEL oscillations were evaluated and it was confirmed that the FEL oscillated at the order of the harmonic with the higher effective gain. The ratio between the effective gain for the fundamental and that for the third harmonic was controlled by the dispersive gap. It was also demonstrated that a spectral measurement of the FEL-based Compton scattering X-ray beam was effective for directly observing the switching of the harmonic order. These results contribute to the development of higher-harmonic FEL oscillations suppressing the fundamental FEL oscillation in the extreme ultraviolet and X-ray regions.

## Abstract 2

Switching of the harmonic order in free-electron laser oscillations by adjusting the dispersive gap of the optical klystron is demonstrated.

## 1. Introduction   

The use of higher-harmonic oscillations in free-electron lasers (FELs) is an effective technique for extending the lasing wavelength to the shorter-wavelength region (Colson, 1981 ▸). Short-wavelength coherent light sources can be obtained with a lower-energy electron beam using the higher harmonics, and the technique based on the higher-harmonic oscillations is employed for the development of self-amplified spontaneous emission (SASE) FELs (Schneidmiller & Yurkov, 2012 ▸) and high-gain harmonic generation FELs (Yu *et al.*, 2000 ▸; Abo-Bakr *et al.*, 2008 ▸; Zhao *et al.*, 2018 ▸) in the extreme ultraviolet and X-ray regions. For resonator-type FELs, X-ray FEL oscillators using higher harmonics were proposed with an electron beam energy smaller than 4 GeV (Dai *et al.*, 2012 ▸; Deng & Dai, 2013 ▸). We have developed a resonator-type higher-harmonic FEL with the infrared FEL device in the storage ring NIJI-IV (Sei *et al.*, 2010 ▸). The seventh harmonic oscillation, which is the highest order of the harmonic FEL oscillations, was achieved with the infrared FEL device of NIJI-IV at wavelengths around 890 nm (Sei *et al.*, 2012*a* ▸). Moreover, we have reported the characteristics of the higher-harmonic FEL and its effect on the electron beam in detail (Sei *et al.*, 2014 ▸, 2017*a* ▸).

To select the order of the harmonic in the resonator-type FEL devices, conventionally, an aperture or etalon was inserted into the optical cavity (Benson & Madey, 1989 ▸; Hajima *et al.*, 2001 ▸; Kubarev *et al.*, 2011 ▸). These additional elements controlled the effective gain for each harmonic FEL by changing the condition of the optical cavity. However, they also increased the cavity loss with the selection of the harmonic. These techniques could not be employed in SASE FELs to select the order of the harmonic.

Therefore, we attempted to select the order of the harmonic in an FEL oscillation by adjusting a parameter of the undulator which changed the balance between the effective gains of the FEL oscillations with different harmonic orders. The Duke FEL system has successfully switched the order of the harmonic FEL using multiple optical klystrons (Wu *et al.*, 2015 ▸). However, using a simpler approach, even one optical klystron can make the effective gain of the higher-harmonic FEL larger than that of the fundamental FEL by controlling the dispersive section. As these techniques do not affect the optical cavity, it is simple to switch the oscillation harmonics. Moreover, experimental results of phase control between the undulator sections will provide a better understanding of not only the resonator-type FEL but also the SASE FEL. In this study, we describe the infrared FEL device in NIJI-IV, which realizes various higher-harmonic FELs, and we evaluate the effective gains for the fundamental and third-harmonic FELs when the dispersive gap of the optical klystron is changed. In addition, the switching of the order of the harmonic FEL by controlling the dispersive gap is demonstrated.

## 2. Infrared FEL device in the storage ring NIJI-IV   

Although the storage ring NIJI-IV is compact with a circumference of 29.6 m, it has two 7.25 m straight sections (Yamazaki *et al.*, 1993 ▸). The radio-frequency cavity is operated at a frequency of 162.1 MHz, and the maximum number of electron bunches stored in NIJI-IV is 16. A planar optical klystron is installed in each straight section, and an ultraviolet FEL device with the optical klystron ETLOK-II and an infrared FEL device with the optical klystron ETLOK-III have been constructed in the southern and northern straight sections, respectively (Sei *et al.*, 2002 ▸). Short-wavelength FELs were developed with ETLOK-II in the wavelength region 198–595 nm (Yamada *et al.*, 2004 ▸).

The infrared FEL device is composed of ETLOK-III and an optical cavity with a length of 14.8 m, which is equal to half of the circumference of NIJI-IV. ETLOK-III has two 1.4 m-long undulator sections consisting of seven periods and a 0.72 m dispersive section. The gap of the undulator section, *g*_u_, can be adjusted from 36 mm to 150 mm, and the maximum deflection parameter *K* is 10.4. Although the gap of the dispersive section, *g*_d_, can be adjusted in the range 42 mm to 188 mm, it can be at most only 38 mm wider than *g*_u_. However, *g*_d_ can be changed independently of *g*_u_; therefore, the effective gain and output power of the FEL can be controlled by *g*_d_ while the lasing wavelength is fixed. In infrared FEL experiments, the electron-beam energy was set to 310 MeV (Sei *et al.*, 2009 ▸). The energy spread of the electron beam in a single-bunch operation was 4 × 10^−4^ in the beam-current region of 5 mA or less. The electron-beam sizes are 0.9 mm in the horizontal direction and 0.2 mm in the vertical direction at the centre of ETLOK-III. The fundamental and higher-harmonic FELs oscillated over a wide wavelength region of 549–2683 nm, obtained with the infrared FEL device in NIJI-IV (Sei *et al.*, 2010 ▸, 2012*b* ▸). We achieved FEL oscillations up to the seventh harmonic, which is the highest achieved order in higher-harmonic FEL oscillations (Sei *et al.*, 2012*a* ▸). The main parameters of the infrared FEL device in NIJI-IV are arranged in Table 1 ▸.

**Figure 1:**
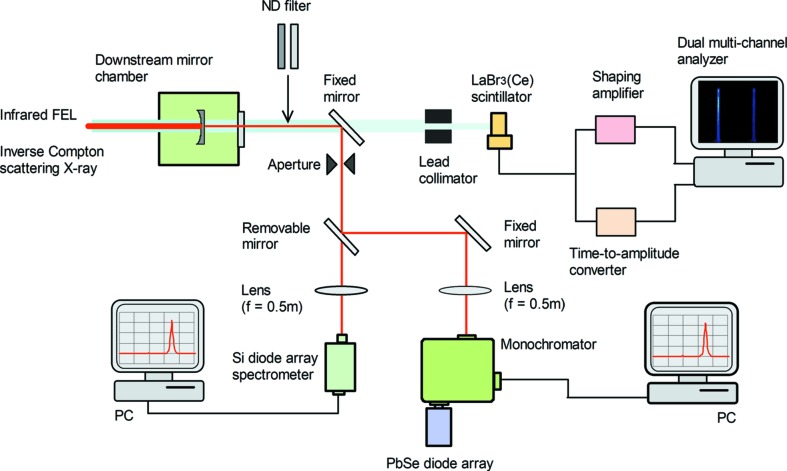
Measurement system at the downstream side of the infrared FEL device in the storage ring NIJI-IV. Using a removable mirror, the optical path could be switched between the near- and mid-infrared detectors.

The wavelength of the FEL oscillation was measured in the atmosphere using a light beam transmitted from a dielectric multilayer mirror located at the downstream side of the optical cavity. The measurement system at the downstream side of the infrared FEL device is shown in Fig. 1 ▸. The spectrum of the light beam was measured by a Si photodiode array spectrometer for a wavelength region of 0.8−1.1 µm and a PbSe photodiode array attached at a monochromator for a wavelength region of 2−5 µm. The spectral resolutions were 0.83 nm for the Si photodiode array spectrometer and 1.3 nm for the PbSe photodiode array. The sensitivity of the mid-infrared detector is as low as 1 nW per channel, and it was marginal to observe the FEL spectrum on the lasing threshold. In order to prevent dimming by dividing the optical path, we did not simultaneously observe in the near- and mid-infrared regions. An FEL-based Compton scattering X-ray beam transmitted through a plane mirror passed through a lead collimator with a bore diameter of 10 mm and thickness of 100 mm (Ogawa *et al.*, 2012 ▸; Sei *et al.*, 2017*b* ▸). The inverse Compton scattering X-ray beam passing through the lead collimator was measured using a LaBr_3_(Ce) scintillation detector with a diameter of 25 mm and length of 51 mm. The temporal resolution of this detector was 1.3 ns. This value was sufficiently smaller than the interval between the electron bunches in the full-bunch operation; therefore, we could distinguish the inverse Compton scattering X-ray pulse generated by each electron bunch.

## 3. FEL gain for higher harmonics of the ETLOK-III   

For a planar undulator, odd harmonics of the spontaneous emission are also radiated on the axis of the electron-beam orbit in the undulator, and they amplify by interacting with the electron beam (Colson, 1981 ▸). The resonant wavelength of the *n*th harmonic for the planar undulator, *λ_n_*, is
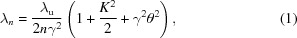
where λ_u_, *γ* and *θ* are the undulator period, electron energy in units of its rest mass and observation angle with respect to the axis of the electron-beam orbit, respectively. In this study, we consider only the behaviour of the FEL oscillations on the axis of the electron beam orbit and, in addition, the order of the harmonic *n* is assumed to be odd. According to the one-dimensional FEL theory (Colson, 1981 ▸), the FEL gain for the *n*th harmonic *G_n_* is expressed by the FEL gain for the fundamental *G*_1_ by
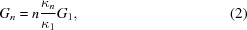


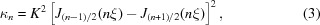


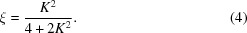



**Figure 2:**
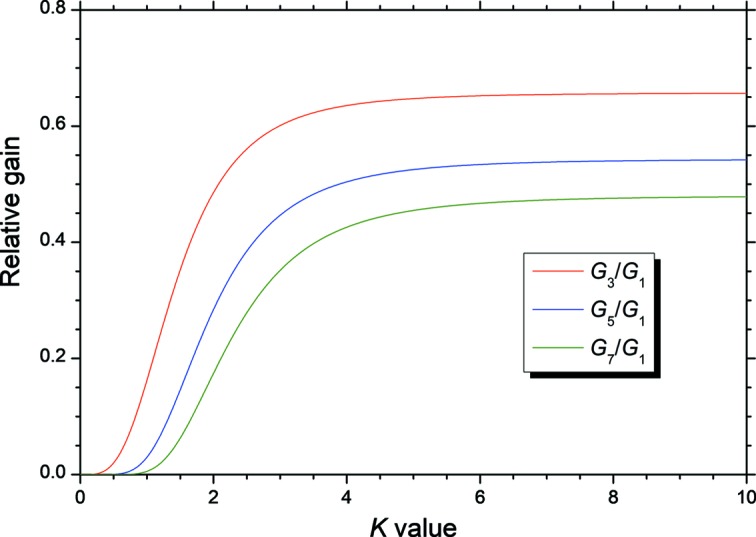
Dependence of the higher-harmonic FEL gains for a planar undulator normalized by the fundamental FEL gain as a function of the *K* value.

Fig. 2 ▸ shows the dependence of the higher-harmonic FEL gain normalized by the fundamental FEL gain (*i.e.* *G_n_*/*G*_1_) as a function of the *K* value (Brau, 1990 ▸; Schneidmiller *et al.*, 2017 ▸). This ratio monotonously increases with the *K* value and saturates to a value smaller than 1. However, the figure indicates that the odd-harmonic FEL gain is not small if the *K* value is sufficiently larger than 1.

**Figure 3:**
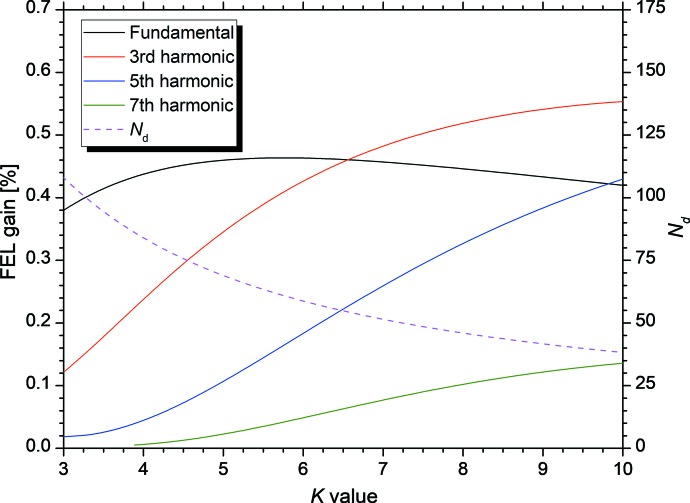
Dependence of *N*_d_ (dotted line) and higher-harmonic FEL gains (solid lines) for the optical klystron ETLOK-III as a function of the *K* value. The dispersive gap was set to the maximum value in the movable range.

The FEL gain for the resonator-type FEL device depends on the filling factor *F*_f_, which can be calculated from the overlap integral between the electron beam and resonated light beam along the optical klystron. Since the spot size of the light beam is smaller as the wavelength of the light beam is shorter, *F*_f_ increases with the order of the higher harmonic. Using equation (2) and the equation for the fundamental FEL gain formulated by Deacon (Deacon *et al.*, 1984 ▸; Elleaume, 1984 ▸), the FEL gain for the higher harmonic of the planar optical klystron, *g*_OK,*n*_, is expressed as
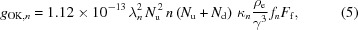
where *N*_u_, *N*_d_, ρ_e_ and *f_n_* are the number of periods in one undulator section, number of periods of the fundamental wavelength passing over an electron in the dispersive section, peak-electron density of a bunch in m^−3^ and the modulation rate for the *n*th harmonic, respectively. Although *f_n_* depends on factors representing the effects of the gain reduction, it is approximated by *f_γn_*, which represents the contribution of the energy spread of the electron bunch *σ_γ_*/*γ* (Sei *et al.*, 2010 ▸),
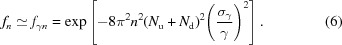



As shown in equations (5) and (6), the higher-harmonic FEL gain is sensitive to the parameter *N*_d_. Although the optimal value of *n*(*N*_u_ + *N*_d_) for the FEL gain was approximately 200 for the infrared FEL device in NIJI-IV (Couprie *et al.*, 1989 ▸; Sei *et al.*, 2010 ▸), ETLOK-III could not satisfy this condition for higher harmonics in the whole range of available *K* values owing to the relationship between *g*_u_ and *g*_d_. Fig. 3 ▸ shows the relationship between the *K* value and lower limit of *N*_d_. It also shows the fundamental and higher-harmonic FEL gains at an electron-beam current of 5 mA and lower limit of *N*_d_ in the single-bunch operation. It is worth noting that the higher-harmonic FEL gain increases with the *K* value owing to the decrease of *N*_d_. In particular, for the third harmonic, *g*_OK__,3_ > *g*_OK__,1_/2 holds in the region *K* > 4. When the cavity loss at the fundamental wavelength is set to be larger than half of that at the third-harmonic wavelength, it is possible to select either the fundamental FEL oscillation or third-harmonic FEL oscillation by adjusting *g*_d_. We performed experiments on the control of the harmonic order in the FEL oscillations in the region *K* > 4.

## 4. Switching of the harmonic order in FEL oscillations   

**Figure 4:**
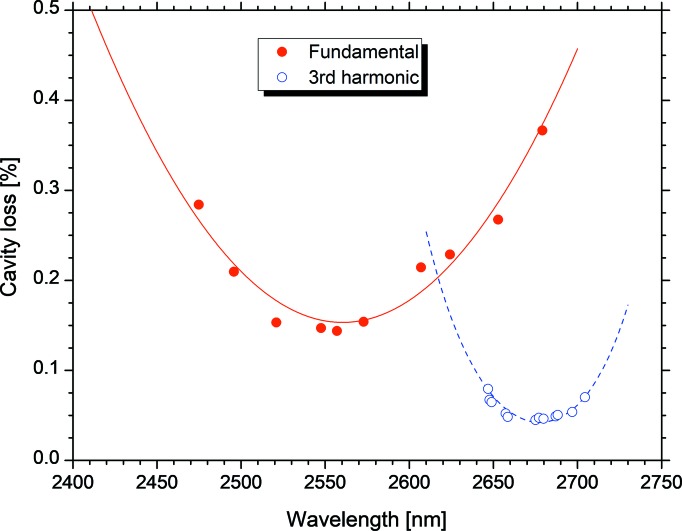
Measured cavity losses for the first (red solid circles) and third (blue open circles) reflectivity curves. The red and blue curves represent the calculated cavity losses for the first and third reflectivity curves considering the complex dielectric constant of the Nb_2_O_5_/SiO_2_ multilayer mirrors and carbon contamination, respectively (Sei *et al.*, 2012*c* ▸). Note that the third reflectivity curve is plotted for three times the wavelength.

Using the infrared FEL device in NIJI-IV, the third-harmonic FEL oscillations were achieved at *K* values in the range 3.19–5.62. In the region of high *K* values, the ratio of the third-harmonic FEL gain to the fundamental FEL gain is larger, and it is simple to control the order of the harmonic in the FEL oscillation. However, it becomes challenging to obtain high-reflectivity mirrors at wavelengths larger than 3 µm. Therefore, the *K* value was set to around 4.2 and the fundamental wavelength was approximately 2.65 µm, where high-reflectivity dielectric multilayer mirrors were available. The reflectivities of these mirrors around the wavelength of the third harmonic were high owing to the higher reflectivity of the dielectric multilayer (Sei *et al.*, 2011 ▸). The cavity loss of a pair of Nb_2_O_5_/SiO_2_ multilayer mirrors used in the experiments is shown in Fig. 4 ▸. The wavelengths of the cavity loss for the third reflectivity are tripled for comparison. Fig. 4 ▸ illustrates that, as the refractive indices of Nb_2_O_5_ and SiO_2_ are larger at shorter wavelengths in the infrared region (Edlinger *et al.*, 1989 ▸), the high-reflectivity region for the third reflectivity shifts to longer wavelengths compared with one-third of the target wavelength of the Nb_2_O_5_/SiO_2_ multilayer mirrors. As already reported (Sei *et al.*, 2012*c* ▸), it was possible to switch the order of the harmonic in the FEL oscillation by adjusting the *K* value. However, it was more important to switch the order by adjusting *g*_d_ without changing the lasing wavelength. Either the fundamental or third-harmonic FEL oscillated at *K* values in the range 4.16–4.27.

**Figure 5:**
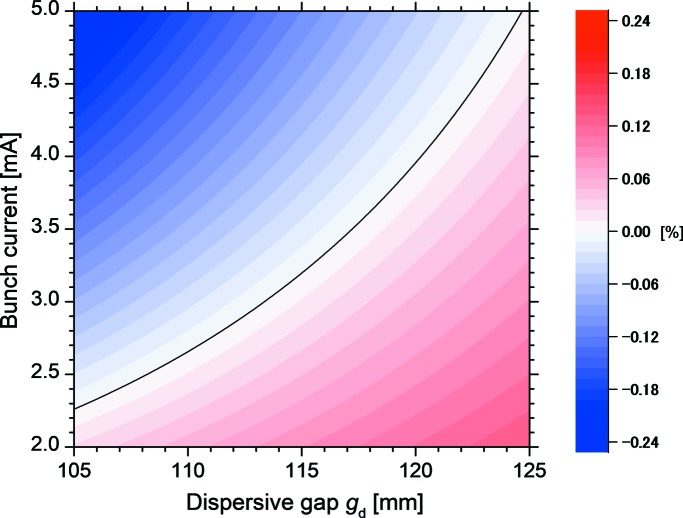
Calculated two-dimensional (dispersive gap and bunch current) mapping of the difference between the effective gain of the third harmonic and that of the fundamental. The solid curve represents the threshold condition to switch between the fundamental and third-harmonic FEL oscillations.

**Figure 6:**
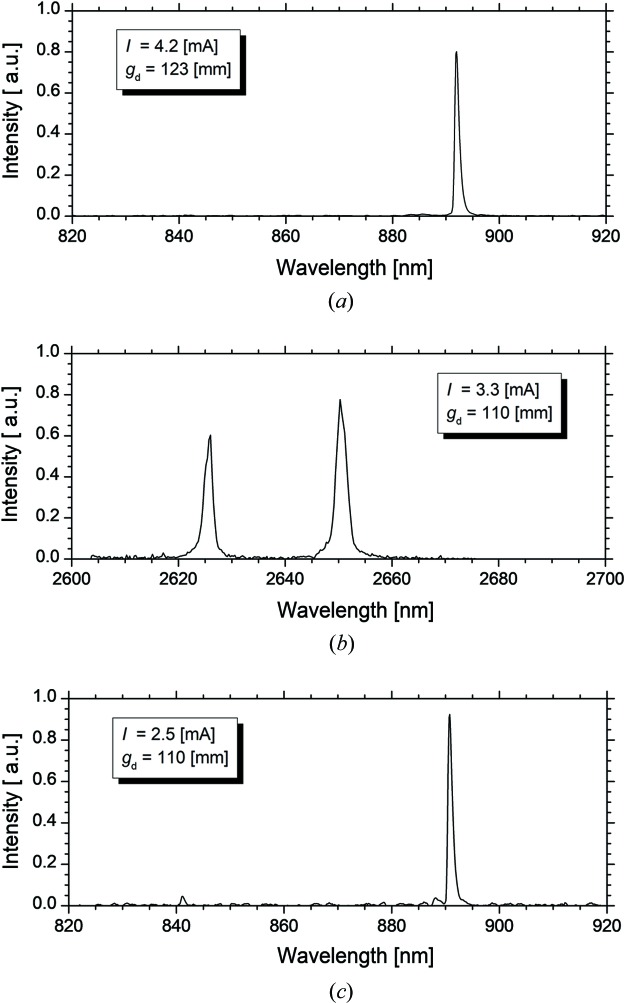
Observed transition of the infrared FEL spectra. The electron-beam current *I* and *g*_d_ were 4.2 mA and 123 mm in (*a*), 3.3 mA and 110 mm in (*b*) and 2.5 mA and 110 mm in (*c*), respectively.

Therefore, the *K* value was set to 4.19 in the experiments for the control of the order of the harmonic in the FEL oscillation. The cavity losses for the fundamental and third harmonic were 2.7 × 10^−3^ at a wavelength of 2.65 µm and 4.5 × 10^−4^ at a wavelength of 893 nm, respectively (Sei *et al.*, 2012*c* ▸). As the cavity loss at wavelengths around 893 nm was significantly lower than that at wavelengths around 2.65 µm, it was expected that only the third-harmonic FEL would oscillate in the region of low electron-beam currents. On the other hand, the fundamental FEL gain was higher than the third-harmonic FEL gain at a *K* value of 4.19; the order of the harmonic in the FEL oscillation would be switched from the third to the fundamental at a certain electron-beam current. This threshold value of the electron-beam current was evaluated by comparing the effective gain, *i.e.* the difference between the FEL gain and cavity loss. Fig. 5 ▸ shows the difference, obtained by subtracting the effective gain for the third harmonic from that for the fundamental, as a function of the electron-beam current per bunch and *g*_d_. As expected, the difference became larger and the threshold current became higher with the increase of *g*_d_. We then changed *g*_d_ from 123 mm to 110 mm while the third-harmonic FEL was oscillating at an electron-beam current of 4.2 mA in the single-bunch operation. The electron-beam current decreased from 4.0 mA to 3.4 mA with the change in *g*_d_. As shown in Fig. 6 ▸, by changing the near-infrared detector to the mid-infrared detector, it was confirmed that the third-harmonic FEL oscillated at a *g*_d_ of 123 mm and that the fundamental FEL oscillated at a *g*_d_ of 110 mm. As the effective gain at a wavelength of 2.65 µm was as low as 0.14%, the FEL using the optical klystron oscillated in two longitudinal modes as shown in Fig. 6 ▸(*b*). When the electron-beam current decreased below the threshold, the effective gain for the third harmonic could exceed that for the fundamental. Only the third-harmonic FEL oscillated at the electron-beam current of 2.5 mA and *g*_d_ of 110 mm. Therefore, we could switch between the fundamental and third harmonic in the FEL oscillations by adjusting the dispersive gap.

**Figure 7:**
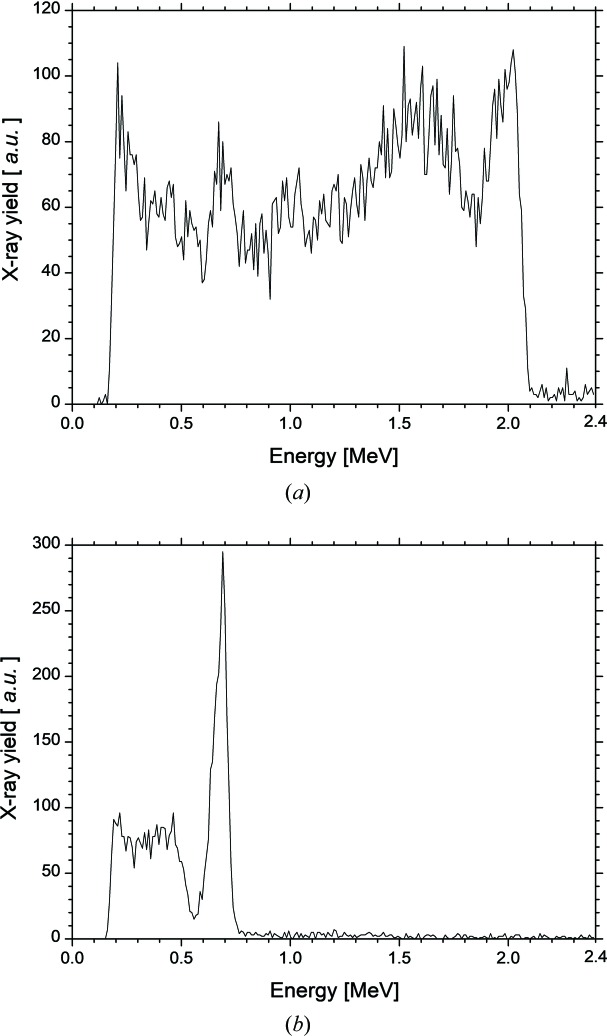
Measured spectra of the FEL-based Compton scattering X-ray beam generated by the electron bunch #0. The dispersive gap was 123 mm in (*a*) and 112 mm in (*b*). The currents of the electron bunches #0, #5 and #10 were 3.3 mA, 3.6 mA and 4.5 mA in (*a*) and 2.7 mA, 2.8 mA and 3.2 mA in (*b*), respectively.

As our measurement system could not simultaneously observe the FEL spectra in the near- and mid-infrared regions, the switching of the order of the harmonic in the FEL oscillation was not directly observed through the FEL spectrum. Therefore, we measured spectra of the inverse Compton scattering X-rays and attempted to observe the harmonic order stored in the optical cavity. In order to generate FEL-based Compton scattering X-ray beams, we filled RF-buckets #0, #5 and #10 with bunch intervals of 31 ns, 31 ns and 37 ns, respectively (Sei *et al.*, 2017*b* ▸). The electron bunches #0 and #5 did not contribute to the FEL oscillations owing to coupled-bunch instability, and only electron bunch #10 achieved the FEL oscillation in these experiments. Fig. 7 ▸(*a*) shows the measured spectrum of the FEL-based Compton scattering X-ray beam generated by the electron bunch #0 at a current of 3.3 mA bunch^−1^. The current of the electron bunch #10 and *g*_d_ were 4.5 mA bunch^−1^ and 123 mm, respectively. The total yield of the inverse Compton scattering X-ray beam was 3 × 10^4^ photons s^−1^ considering the detection efficiency of the detector system (Ogawa *et al.*, 2012 ▸). It is worth noting that a full-energy peak and a small peak were observed at energies of 2.0 MeV and 0.69 MeV, respectively. The full-energy peak indicates that the FEL was oscillating at the third harmonic, whereas the small peak, whose yield was approximately 3% of that of the FEL-based Compton scattering X-ray beam, indicates that the resonant fundamental in the optical cavity did not reach the FEL oscillation. Fig. 5 ▸ supposes that only the third-harmonic FEL oscillated in the experimental condition. When the current of electron bunch #10 was 3.2 mA bunch^−1^, *g*_d_ was changed to 112 mm. The current of electron bunch #0 was 2.7 mA. The total yield of the inverse Compton scattering X-ray beam under this experimental condition was 2 × 10^4^ photons s^−1^. Fig. 7 ▸(*b*) indicates that the order of the harmonic in the FEL oscillation switched from the third to the first. The switching of the order by adjusting the dispersive gap was directly demonstrated with a spectral measurement of the FEL-based Compton scattering X-ray beam.

## 5. Discussion and conclusions   

Using the infrared FEL device in the storage ring NIJI-IV, we demonstrated that the order of the harmonic in the FEL oscillation could be switched by adjusting the dispersive gap of the optical klystron without changing the characteristics of the optical cavity. The FEL gain of the higher harmonic was formulated for a planar optical klystron. The effective gains for the fundamental and third-harmonic FEL oscillations were evaluated, and the condition that both effective gains were equalized was derived as a curve on a two-dimensional graph with axes of the electron-beam current per bunch and dispersive gap. When the dispersive gap was adjusted across the curve, the switching of the order was confirmed by the spectral measurement of the infrared FEL oscillations in the single-bunch operation. Moreover, we directly demonstrated the switching of the order by the spectral measurement of the FEL-based Compton scattering X-ray beam in the multi-bunch operation. It was possible to oscillate the higher-harmonic FEL while suppressing the oscillation of the fundamental FEL without the optical cavity. Our results could be useful for the development of higher-harmonic FEL oscillations with a lower-energy electron beam in the extreme ultraviolet and X-ray regions (Li & Deng, 2017 ▸).

For the resonator-type FEL devices, the cavity loss for each harmonic order can be controlled using dielectric multilayer mirrors. By adjusting the current for each electron bunch, it is possible to oscillate FELs at multiple wavelengths in one optical cavity. Our results contribute to realizing the multi-wavelength FEL device.

## Figures and Tables

**Table 1:** Main parameters of the infrared FEL device in the storage ring NIJI-IV. The electron-beam parameters were measured at a current of 5 mA in the single-bunch operation

Storage ring (NIJI-IV)	Circumference (m)		29.6
	Straight section	Length (m)	7.25
	Revolution frequency (MHz)		162.1
	Maximum number of bunches		16
	Energy (MeV)		310
	Energy spread		4 × 10^−4^
	Beam size (mm)	Horizontal	0.9
		Vertical	0.2

Optical cavity	Length (m)		14.8
	Mirror diameter (mm)		≤50.8

Optical klystron (ETLOK-III)	Undulator section	Period (m)	0.2
		Period number	7 + 7
		Gap (*g*_u_) (mm)	36–150
		Maximum *K* value	10.4
	Dispersive section	Length (m)	0.72
		Gap (*g*_d_) (mm)	42–188
